# Priority Intervention Targets Identified Using an In-Depth Sampling HIV Molecular Network in a Non-Subtype B Epidemics Area

**DOI:** 10.3389/fcimb.2021.642903

**Published:** 2021-03-29

**Authors:** Bin Zhao, Wei Song, Minghui An, Xue Dong, Xin Li, Lu Wang, Jianmin Liu, Wen Tian, Zhen Wang, Haibo Ding, Xiaoxu Han, Hong Shang

**Affiliations:** ^1^ NHC Key Laboratory of AIDS Immunology (China Medical University), National Clinical Research Center for Laboratory Medicine, The First Affiliated Hospital of China Medical University, Shenyang, China; ^2^ Laboratory Medicine Innovation Unit, Chinese Academy of Medical Sciences, Shenyang, China; ^3^ Key Laboratory of AIDS Immunology of Liaoning Province, Shenyang, China; ^4^ Collaborative Innovation Center for Diagnosis and Treatment of Infectious Diseases, Hangzhou, China; ^5^ Department of Food Safety and Nutrition, Shenyang Center for Health Service and Administrative Law Enforcement (Shenyang Center for Disease Control and Prevention), Shenyang, China

**Keywords:** HIV-1, molecular networks, genetic distance threshold, molecular clusters, sociodemographic characters

## Abstract

Molecular network analysis based on the genetic similarity of HIV-1 is increasingly used to guide targeted interventions. Nevertheless, there is a lack of experience regarding molecular network inferences and targeted interventions in combination with epidemiological information in areas with diverse epidemic strains of HIV-1.We collected 2,173 *pol* sequences covering 84% of the total newly diagnosed HIV-1 infections in Shenyang city, Northeast China, between 2016 and 2018. Molecular networks were constructed using the optimized genetic distance threshold for main subtypes obtained using sensitivity analysis of plausible threshold ranges. The transmission rates (TR) of each large cluster were assessed using Bayesian analyses. Molecular clusters with the characteristics of ≥5 newly diagnosed cases in 2018, high TR, injection drug users (IDUs), and transmitted drug resistance (TDR) were defined as priority clusters. Several HIV-1 subtypes were identified, with a predominance of CRF01_AE (71.0%, 1,542/2,173), followed by CRF07_BC (18.1%, 393/2,173), subtype B (4.5%, 97/2,173), other subtypes (2.6%, 56/2,173), and unique recombinant forms (3.9%, 85/2,173). The overall optimal genetic distance thresholds for CRF01_AE and CRF07_BC were both 0.007 subs/site. For subtype B, it was 0.013 subs/site. 861 (42.4%) sequences of the top three subtypes formed 239 clusters (size: 2-77 sequences), including eight large clusters (size **≥**10 sequences). All the eight large clusters had higher TR (median TR = 52.4/100 person-years) than that of the general HIV infections in Shenyang (10.9/100 person-years). A total of ten clusters including 231 individuals were determined as priority clusters for targeted intervention, including eight large clusters (five clusters with≥5 newly diagnosed cases in 2018, one cluster with IDUs, and two clusters with TDR (K103N, Q58E/V179D), one cluster with≥5 newly diagnosed cases in 2018, and one IDUs cluster. In conclusion, a comprehensive analysis combining in-depth sampling HIV-1 molecular networks construction using subtype-specific optimal genetic distance thresholds, and baseline epidemiological information can help to identify the targets of priority intervention in an area epidemic for non-subtype B.

## Introduction

Molecular epidemiology analysis with viral sequences among HIV-1-infected patients has long been used to explore the origin of HIV and to track the course of HIV spread (Aldous et al., 2012; Wertheim et al., 2014; Wertheim et al., 2017).In recent years, many studies inferred HIV-1 transmission clusters among populations using phylogenetic (Ragonnet-Cronin et al., 2013) or genetic distance-based methods (Kosakovsky Pond et al., 2018). Some studies even used these methods to inform near real-time interventions for HIV-1 infections (Pasquale et al., 2018). On-line tools for molecular cluster inference are available, including the HIV Transmission Cluster Engine (HIV-TRACE) (Kosakovsky Pond et al., 2018), a simplified genetic distance (GD)-based program developed by Kosakovsky Pond, that rapidly processes tens of thousands of data to infer potential HIV transmission clusters among a population. The partial HIV-1 *pol* gene is most commonly used for molecular network analysis because of the rich data from HIV drug resistance testing worldwide (Hassan et al., 2017). Most transmission network studies have been conducted in the USA (Little et al., 2014; Wertheim et al., 2017; Prevention and Prevention, 2017) and Europe (Chaillon et al., 2017; Stecher et al., 2018; Chaillon et al., 2019), where subtype B acts as the predominant strain and has shown a rather stable transmission rate in recent years (Hightower et al., 2013).

By the end of 2018, approximately 1.25 million people were living with HIV in China (Lyu and Chen, 2019). China is plagued with complex HIV-1 strains. According to the latest National HIV Molecular Epidemiological Survey, 18 known HIV subtypes were detected, predominant of four subtypes, including CRF07_BC(41.9%), CRF01_AE(33.2%), CRF08_BC(10.9%), and subtype B (4.0%). Among men who have sex with men (MSM), CRF07_BC and CRF01_AE were all over 40%, while subtype B and new recombinants were all 5.6% (Zhong, 2019). These findings suggest that the HIV epidemic situation in China is quite different from those of the USA and Europe. Recently, several studies in China have inferred the HIV molecular networks of CRF01_AE using publicly available sequences around China (Wang et al., 2019), local MSM population (Chen et al., 2018; Yan et al., 2020), and patients suffering from virologic failure (Yuan et al., 2019). Nevertheless, these studies usually use a phylogenetic approach or follow the genetic distance threshold of subtype B and the sampling depth remains low. More importantly, there is a lack of molecular networks combined with epidemiological information to guide the accurate targeting of the intervention population.

Shenyang city is reported to have the second-largest MSM population in China (after Beijing) with an estimated 140,000 MSM (DH and J, 2006). In the last 10 years, MSM in Shenyang was the main severely affected population, accounting for more than 80% of new HIV infections and CRF01_AE and CRF07_BC were the most prevalent strains (An et al., 2012; Han et al., 2013; Zhao et al., 2015). Moreover, there were close links between MSM in Shenyang and other regions in China according to previous reports (Han et al., 2013; Han et al., 2015). In the present study, molecular networks of major HIV pandemic strains were constructed retrospectively among over 90% of newly diagnosed patients between 2016-2018 in Shenyang, where diverse HIV subtypes and circulating recombination forms (CRFs) have been reported among the various high-risk populations (Zhao et al., 2011; Zhao et al., 2015). A transmission network study of the whole HIV-infected population combined with epidemiological data in Shenyang might offer experiences regarding transmission network-based targeted prevention in areas experiencing complex HIV-1 epidemics.

## Materials and Methods

### Study Population

A retrospective molecular epidemiology study was conducted in Shenyang city. Between 2016 and 2018, a total of 2,577 HIV patients living in Shenyang were newly diagnosed, from which 2,354 (91.3%, 2,354/2,577) cryopreserved HIV-positive plasma samples at the time of diagnosis were available for drug resistance detection and HIV-1 LAg-Avidity EIA. All participants were HIV treatment-naïve at the time of sample collection. The corresponding demographic data were collected simultaneously. The study was approved by the Institutional Review Board of China Medical University. Recent HIV infections (RHI) were determined using records of seroconversion within 6 months (Han et al., 2011) or based on the results of a limiting antigen avidity enzyme immunoassay (HIV-1 LAg-Avidity EIA).

### Detecting of HIV-1 LAg-Avidity EIA

ELISA tests for distinguishing RHI from chronic HIV infection (CHI) were performed using HIV-1 LAg-Avidity EIA (Maxim Biomedical, 2013, Bethesda, MD, USA), according to the manufacturer’s instructions using serum and dried blood spots. If the optical density (OD) value≤ 2.0, triplicate ‘confirmatory’ testing was required. In the confirmatory test, if the OD value ≤1.5, the sample was considered to be RHI as the final results.

### Subtyping, Phylogenetic, and Genotypic Resistance Analyses

The HIV *pol* gene (HXB2: 2,253-3,318) was amplified and sequenced for drug resistance detection using a previously published method (Stecher et al., 2018). Only the first sequence was selected if more than one sequence was available for a participant. The subtype of each sequence was determined using the Maximum-Likelihood phylogeny analysis using Mega 7.0.14 (Kumar et al., 2016). All reference sequences were downloaded from the Los Alamos database (LANL). Maximum-Likelihood phylogenetic trees (GTR nucleotide substitution model) were constructed using the SH-aLRT and the ultrafast bootstrap in IQ-TREE (Nguyen et al., 2015) with 1,000 replicates. Trees were visualized using Figtree (version 1.4.2) (http://beast.bio.ed.ac.uk). The clusters with SH-aLRT >90 (Anisimova et al., 2011) and ultrafast bootstrap values >90 were considered as a lineage (Feng et al., 2013). To identify the potential recombinants, candidate sequences were analyzed using the Recombination Identification Program (RIP) v3.0 (https://www.hiv.lanl.gov/content/sequence/RIP/RIP.html).

Transmitted drug resistance (TDR) mutations were determined using the online Calibrated Population Resistance Tool (https://hivdb.stanford.edu/cpr/) version 8.8 (Gifford et al., 2009). Sequences were used to identify genetic positions (i.e., codons) of putative drug resistance.

### Molecular Network Analyses

The molecular networks of three main subtypes, CRF01_AE, CRF07_BC, and B were inferred based on the nucleotide GD by HIV-TRACE (www.hivtrace.org) (Kosakovsky Pond et al., 2018). All pairwise distances were computed and a putative linkage between two individuals was considered whenever distance measures between two sequences (the Tamura-Nei 93 substitution model) fell below the GD threshold (Little et al., 2014). Clusters were defined as connected components of the network comprising ≥2 nodes.

To infer the high-resolution networks of the three main HIV-1 subtypes in Shenyang, we applied a sensitivity analysis across the range of epidemiologically plausible GD thresholds, from more conservative (0.001 subs/site) to more liberal thresholds (0.020 subs/site). High resolution was defined as the minimum GD threshold that could identify the maximum number (not size) of clusters in each subtype (Wertheim et al., 2017). We used HIV-TRACE (Kosakovsky Pond et al., 2018) to infer the molecular network using the above range of plausible GD thresholds.

### Estimated Transmission Rate

We applied a transmission rate (TR) formula to analyze the dynamics of the large clusters (n ≥10 sequences) and to estimate HIV transmission efficiency within each large cluster over time. Bayesian molecular clock phylogenetic inference was conducted to estimate the ages of internal nodes within each cluster using BEAST v.2.6.2 (Bouckaert et al., 2019). A series of parameters (TN93 substitution model, a strict molecular clock, and Bayesian skyline prior to 10 million generations) were set as previously described (Oster et al., 2018). The TR was defined from phylogenetic inferences as to the number of transmission events in the cluster, divided by the total HIV-infected person-time (Oster et al., 2018).

$$TR=\frac{transm\;events}{HIV-positive\;person\;time}=\frac{\left(no.\;people\;in\;cluster-1\right)}{\sum (all\;node\;ages)+longest\;node\;age}=XX\;transm/100\;person\;years$$

### Statistical Analyses

Chi-square tests were used to compare the demographic characteristics and clinical data between the different subtypes. Multivariate logistic regression analysis was performed to identify factors associated with the population within the priority clusters. Categorical variables included demographics, HIV risk factors, and HIV-1 subtype. P-values <0.05 were considered statistically significant. The calculations of all statistical tests were performed using SPSS version 25.0. Graph of the parameters used to infer molecular networks were generated using GraphPad Prism 7.04 (GraphPad Software, La Jolla, CA, USA).

## Results

### Demographic Characteristics of the Studied Population

A total of 2,173 (92.3%, 2,173/2,354) *pol* genes were successfully amplified and sequenced. Subtyping results showed the complicated subtype epidemic in Shenyang. CRF01_AE was the dominant subtype (71.0%, 1,542/2,173), followed by CRF07_BC (18.1%, 393/2,173), subtype B (4.5%, 97/2,173), other subtypes (2.6%, 56/2,173) including C, F1, CRF02_AG, CRF06_cpx, CRF08_BC, CRF33_01B, CRF55_01B, CRF59_01B, CRF65_cpx, CRF67_01B, CRF68_01B, CRF85_BC, CRF103_01BC, and some unique recombinant forms (URFs) (3.9%, 85/2,173).

The demographic characteristics of all the subjects, whose *pol* sequences were successfully acquired, were analyzed; 83.6% (1,817/2,173) were self-reported to be MSM, followed by heterosexual transmission (13.6%, 296/2,173), and injection drug users (IDU, 1.6%, 35/2,173); 93.9% (2,041/2,173) were male. The median age was 31 years old (IQR = 25-44 years, range: 1-89 years); 86.7% (1,883/2,173) were of Han ethnicity. More than half of individuals (63.0%, 1,368/2,173) reported being unmarried. A total of 70.8% (1,539/2,173) had senior high school education or above; 66.2% of them were registered in five major districts of Shenyang; 34.6% (751/2,173) were diagnosed during RHI stages (**Table 1**). The demographic characteristics of the three main subtypes (CRF01_AE, CRF07_BC, and B) were also listed and compared (**Table 1**). The individuals infected with CRF01_AE were most likely to be more than 30 years old (60.3%, p = 0.0001). The individuals infected with CRF07_BC were most likely to be unmarried (70.7%, p = 0.002), MSM (89.1%, p = 0.010), and had the highest level of education (senior high school and above, 77.4%, p = 0.013).

**Table 1:** Population characteristics in Shenyang based on different subtypes.

	All* (n=2,173, %)	CRF01_AE (n=1,542, %)	CRF07_BC (n=393, %)	B (n=97, %)	χ^2^	P
**Year of sample**					4.749	0.314
2016	667 (30.7)	471 (30.5)	119 (30.3)	35 (36.1)		
2017	756 (34.8)	555 (36.0)	125 (31.8)	31 (32.0)		
2018	750 (34.5)	516 (33.5)	149 (37.9)	31 (32.0)		
**Gender**					2.810	0.245
Male	2041 (93.9)	1451 (94.1)	374 (95.2)	88 (90.7)		
Female	132 (6.1)	91 (5.9)	19 (4.8)	9 (9.3)		
**Age at enrollment (years)**					18.000	0.0001
<30	920 (42.3)	611(39.6)	197 (50.1)	50 (51.5)		
≥30	1251 (57.6)	930 (60.3)	195 (49.6)	47 (48.5)		
Unknown	2 (0.1)	1 (0.1)	1 (0.3)	0 (0.0)		
**Race/ethnicity**					3.460	0.177
Han	1883 (86.7)	1333 (86.4)	337 (85.8)	90 (92.8)		
Other	289 (13.3)	208 (13.5)	56 (14.2)	7 (7.2)		
Unknown	1 (0.0)	1 (0.1)	0 (0.0)	0 (0.0)		
**Marital status**					12.525	0.002
Unmarried	1368 (63.0)	943 (61.2)	278 (70.7)	63 (64.9)		
Married/Divorced/widower	798 (36.7)	595 (38.6)	114 (29.0)	34 (35.1)		
Unknown	7 (0.3)	4 (0.3)	1 (0.3)	0 (0.0)		
**Education level**					8.662	0.013
Junior high school and below	624 (28.7)	459 (29.8)	88 (22.4)	29 (29.9)		
Senior high school and above	1539 (70.8)	1075 (69.7)	304 (77.4)	68 (70.1)		
Unknown	10 (0.5)	8 (0.5)	1 (0.3)	0 (0.0)		
**Residence**					0.038	0.981
In the city (Five main districts)	1439 (66.2)	1022 (66.3)	263 (66.9)	64 (66.0)		
Suburb (Other districts)	724 (33.3)	514 (33.3)	130 (33.1)	33 (34.0)		
Unknown	10 (0.5)	6 (0.4)	0 (0.0)	0 (0.0)		
**Infection Route**					15.686	0.010
MSM	1817 (83.6)	1284 (83.3)	350 (89.1)	76 (78.4)		
HST	296 (13.6)	210 (13.6)	36 (9.2)	19 (19.6)		
IDU	35 (1.6)	31 (2.0)	4 (1.0)	0 (0.0)		
Other/unknown	25 (1.2)	17 (1.1)	3 (0.8)	2 (2.1)		
**Infection stage at enrollment**					3.204	0.201
Chronic HIV infection	1357 (62.4)	968 (62.8)	238 (60.6)	67 (69.1)		
Recent HIV infection	751 (34.6)	537 (34.8)	145 (36.9)	26 (26.8)		
Unknown	65 (3.0)	37 (2.4)	10 (2.5)	4 (4.1)		

*All the sequences were included CRF01_AE, CRF07_BC, B, other subtypes, and unique recombinant forms.

### Inferring Molecular Networks of Three Major Subtypes

**Figure 1:**
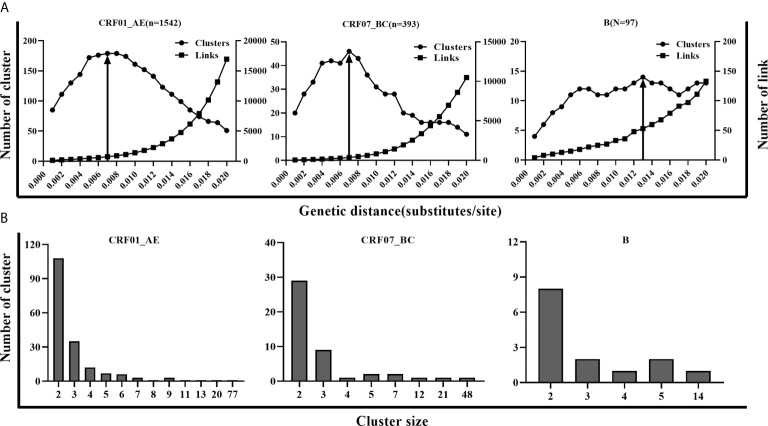
The parameters for inferring molecular network in CRF01_AE, CRF07_BC and B. **(A)** A systematic sensitivity analysis across the range of GD thresholds (0.001 to 0.020 substitutions/site) for 3 main subtypes. The changes in the number of clusters, the number of edges, and maximum cluster size were showed in CRF01_AE, CRF07_BC, and B separately. The line with an arrow denoted the selected optimal GD thresholds for the 3 subtypes. **(B)** Distribution of molecular clusters of three main subtypes along with cluster size in the optimal GD threshold.

Three main subtypes (CRF01_AE, CRF07_BC, and B) accounted for 93.5% (2,032/2,173) of newly diagnosed patients during 2016-2018 in Shenyang city. Considering the different epidemic situations of the three main subtypes in Shenyang, and the highest resolution of molecular networks which could provide the best information to target HIV prevention interventions, we performed a sensitivity analysis across a range of GD thresholds to infer the HIV molecular networks for each of the three main HIV subtypes. For an example of CRF01_AE strains, the most conservative GD of 0.001 subs/site inferred 85 clusters (size range: 2-8), including 199 sequences (cluster rate: 12.9%). By increasing the GD threshold, the number of clusters, cluster size, and the links between clustering persons increased. The maximum cluster number (179, size range: 2-77) was obtained for a GD threshold of 0.007 subs/site. Above this threshold, clusters began to coalesce and the inferred network lost resolution. In other words, the number of inferred clusters began to decrease in conjunction with a sharp increase of clustering individuals (nodes increasing from 617 to 1382) and genetic links (the number of edges increasing from 749 to 16949) (**Figure 1**). Similar to CRF01_AE, 0.007 subs/site was also determined as the optimal GD threshold for CRF07_BC strains, with which the maximum number of clusters of 46 were identified (size: 2-48). However, the optimal GD for subtype B (0.013 subs/site) was much higher than for CRF01_AE and CRF07_BC strains, with which 14 clusters (size: 2-14) were inferred (**Figure 1**). Overall, 861 sequences (42.4%) formed 239 clusters (size: 2-77 sequences), including eight large clusters with more than ten members (four of CRF01_AE clusters, three of CRF07_BC clusters, and one of subtype B cluster). The two largest clusters were the CRF01_AE cluster and CRF07_BC cluster, with member number of 77 and 48, respectively. Patients with subtype B infection had a higher cluster rate (51.5%, 50/97) than did patients with CRF07_BC (49.4%, 194/393) (*p* = 0.700) or CRF01_AE (40.0%, 617/1,542) infection (*p* = 0.026).

The goal of inferring molecular networks is to capture more recent infection events. Therefore, we further explored the performance of the optimal threshold on the identification of RHI in clusters, compared to the GD thresholds (0.005/0.015 subs/site) of B subtype recommended by the guidelines (National Center for HIV/AIDS, JUNE 2018). We found that a slightly lower percentage of RHI were included in clusters with the optimal threshold (0.007 subs/site) than the conservative threshold (0.005 subs/site) for both CRF01_AE and CRF07_BC. However, for subtype B strains, over 10% of RHI was missed with the optimal threshold (0.013 subs/site) than with the strict threshold (0.005 subs/site), but were closed to the performance of the relaxed threshold (0.015 subs/site) (**Table 2**).

**Table 2:** Network parameters with different thresholds for the three main epidemic subtypes in Shenyang.

Subtype	GD threshold(subs/site)	Individuals(cluster rate%)	number of clusters	maximum number of links	median of links (IQR)	Recent HIV infection (%)
CRF01_AE	0.005	494 (32.0)	172	12	1 (1-2)	199 (40.3)
	0.007	617 (40.0)	179	22	2 (1-3)	237 (38.4)
	0.015	1170 (75.9)	99	288	4 (2-9)	403 (34.4)
CRF07_BC	0.005	151 (38.4)	42	15	2 (1-3)	66 (43.7)
	0.007	194 (49.4)	46	29	2 (1-5)	80 (41.2)
	0.015	306 (77.9)	16	185	11 (3-28)	114 (37.3)
B	0.005	24 (24.7)	11	2	1 (1-2)	11 (45.8)
	0.013	50 (51.5)	14	10	2 (1-3)	17 (34.0)
	0.015	53 (54.6)	13	10	2 (1-4)	17 (32.1)

### Estimation of TR for Eight Large Clusters (Size≥10)

Estimation of TR can provide insight into the epidemic dynamics of transmission clusters (Oster et al., 2018). Therefore, we calculated the TR of the clusters with sequence number ≥10. The median TR of eight large clusters (01AE1 to 01AE4, 07BC18 to 07BC20, and B23) was 52.4/100 person-years (IQR 49.0-55.1 years), which was 4.8 times higher than that of the general TR level of Shenyang [10.9/100 person-years (Zhang et al., 2018)] and ten times higher than national estimates in 2018 [4.9/100 person-years (NCAIDS et al., 2018)]. Among the eight large clusters, a TDR cluster (07BC20) had the highest TR of 59.6 per 100 person-years (**Table 3**).

**Table 3:** The demographic characteristics and network-related risk factors of cases in the priority clusters.

Cluster No.	size	Demographic characteristics						Characteristics of active molecular clusters						
		Male (%)	Median age (range)	Han nationality (%)	Unmarried (%)	Education^a^ (%)	In the city (%)	Diagnosis in 2016	Diagnosis in 2017	Diagnosis in 2018	RHI^b^ (%)	TR (py)^c^	Drug-resistant Mutation (%)	Predominant risk factor (%)
01AE1	77	72 (93.5)	35 (16-71)	63 (81.8)	41 (53.2)	45 (58.4)	25 (32.5)	25 (32.5)	36 (46.8)	16 (20.8)	25 (32.5)	55.7		MSM (80.5)
01AE2	20	16 (80.0)	*47 (26-54)*	20 (100.0)	7 (35.0)	10 (50.0)	9 (45.0)	7 (35.0)	4 (20.0)	9 (45.0)	9 (45.0)	48.5		IDU (80.0)
01AE3	13	12 (92.3)	29 (18-56)	8 (61.5)	8 (61.5)	8 (61.5)	8 (61.5)	4 (30.8)	7 (53.8)	2 (15.4)	8 (61.5)	43.5		MSM (84.6)
01AE4	11	9 (81.8)	*44 (20-65)*	9 (81.8)	5 (45.5)	5 (45.5)	2 (18.2)	4 (36.4)	4 (36.4)	3 (27.3)	2 (18.2)	54.9		MSM (81.8)
01AE6	9	7 (77.8)	*46 (36-52)*	9 (100.0)	3 (33.3)	2 (22.2)	4 (44.4)	3 (33.3)	3 (33.3)	3 (33.3)	4 (44.4)			IDU (66.7)
01AE12	6	6 (100.0)	31.5 (23-44)	5 (83.3)	4 (66.7)	4 (66.7)	2 (33.3)	1 (16.7)	0 (0.0)	5 (83.3)	2 (33.3)			MSM (100.0)
07BC18	48	45 (93.8)	34 (18-61)	45 (93.8)	26 (54.2)	35 (72.9)	17 (35.4)	16 (33.3)	13 (27.1)	19 (39.6)	17 (35.4)	53.7		MSM (87.5)
07BC19	21	21 (100.0)	24 (19-49)	18 (85.7)	20 (95.2)	20 (95.2)	10 (47.6)	6 (28.6)	11 (52.4)	4 (19.0)	10 (47.6)	51.1	Q58E/V179D (100.0)	MSM (100.0)
07BC20	12	12 (100.0)	25 (22-36)	10 (83.3)	9 (75.0)	10 (83.3)	8 (66.7)	1 (8.3)	5 (41.7)	6 (50.0)	8 (66.7)	59.6	K103N (66.7)	MSM (91.7)
B23	14	14 (100.0)	29.5 (19-45)	14 (100.0)	12 (85.7)	12 (85.7)	3 (21.4)	5 (35.7)	4 (28.6)	5 (35.7)	3 (21.4)	49.1		MSM (85.7)

^a^Senior high school and above, ^b^Recent HIV infection, ^c^Transmission rate (person-years).

### Priority Intervention Clusters Identified by Molecular Networks Construction Combined With Sociodemographic Information

**Figure 2:**
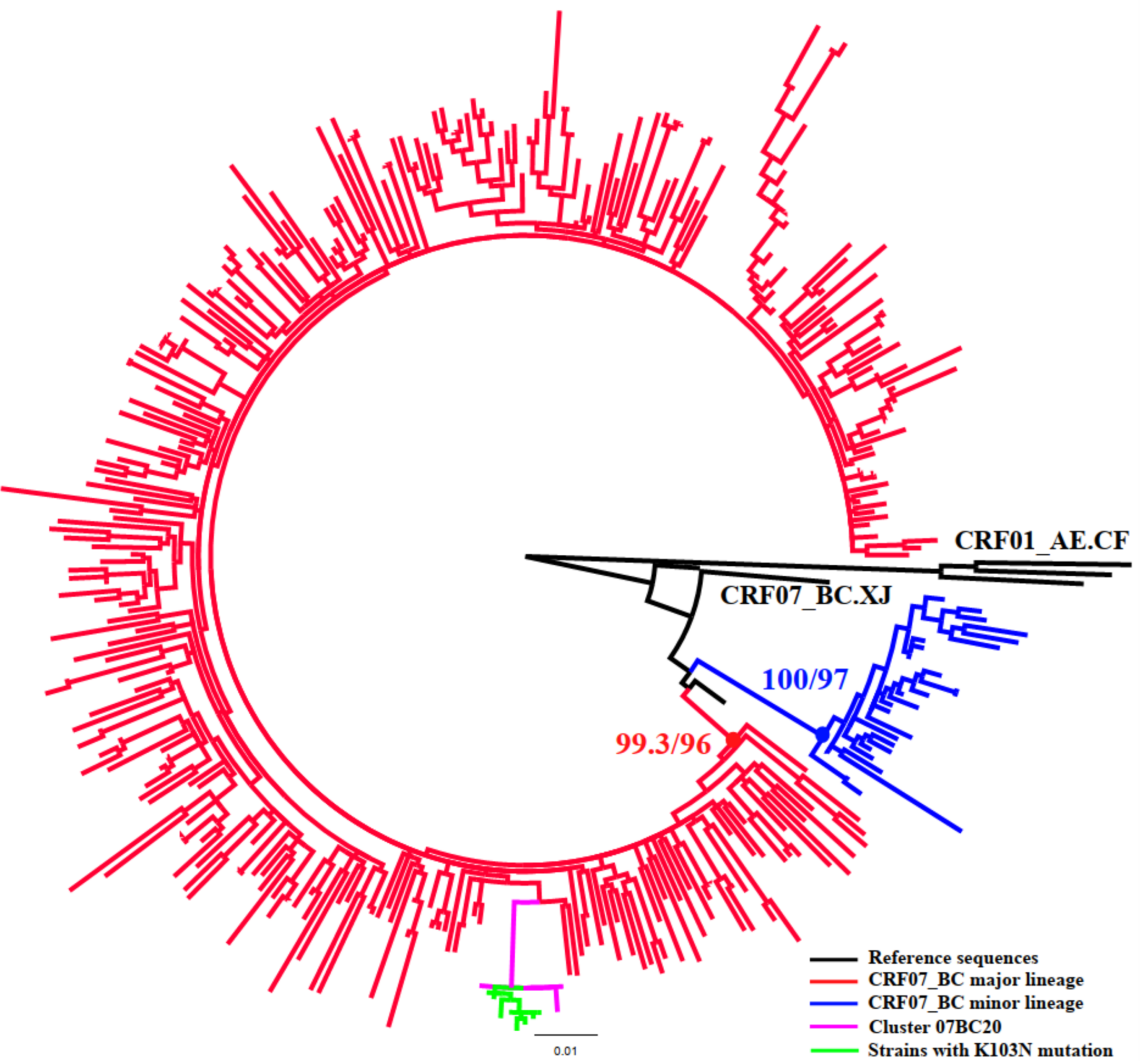
Phylogenetic analysis of subtype CRF07_BC. The phylogenetic tree was constructed using the maximum-likelihood method based on the pol region (HXB2: 2,253 to 3,300nt). HIV-1 subtype CRF01_AE was chosen as an out-group in the CRF07_BC rooted tree. Only the sequences within the two lineages of CRF07_BC (88.8%, 349/393) were included in this ML tree. The molecular cluster (pink) including the strains with K103N (green) located in the main lineage of CRF07_BC (red).

To identify targets for intervention, we used some risk factors related to active transmission clusters (at least five new diagnosed cases in the cluster in the most recent 12-month period (National Center for HIV/AIDS, JUNE 2018) or high TR (Oster et al., 2018), poor outcomes clusters [such as IDU and TDR, etc. (National Center for HIV/AIDS, JUNE 2018; Prevention and center, 2019)]. According to the results above, all eight larger clusters having high TR should be included as a priority for targeted intervention, which contained five clusters with ≥5 newly diagnosed cases in 2018 (01AE1,01AE2, 07BC18, 07BC20, B23), one cluster (01AE2) with IDUs, and two clusters with TDR (07BC19: Q58E/V179D, 100% and 07BC20: K103N, 66.7%, **Figure 2**). one cluster (01AE12) with ≥5 newly diagnosed cases in 2018 and one IDUs cluster (01AE6) were also identified as priority intervention clusters. In summary, a total of ten clusters (cluster 01AE1-4, 01AE6, 01AE12, 07BC18-20, and B23) including 231 individuals were identified as priority intervention targets. The characteristics of the clusters (size >5) including priority intervention clusters are shown in **Table 3**.

## Discussion

We determined the priority clusters for targeted interventions in an area with multiple HIV-1 strain epidemics using HIV molecular network inference with deep sampling sequences and optimized GD thresholds.

It has been noted that the sampling depth is a key factor for the high resolution of transmission network inference; low sampling coverage might lead to missing the potential linkages among infections (Little et al., 2014), and even missing some important small molecular clusters with transmission potential. For example, in the present study, some of the eight cases carrying K103N mutations in cluster 07BC20 might have been missed. Therefore, at least 60% coverage of persons with diagnosed HIV infection is recommended in the guidelines (National Center for HIV/AIDS, JUNE 2018). In this study, *pol* sequences were obtained from 84% (2,173/2,577) of newly diagnosed HIV-infected cases during the study period, much higher than most molecular network studies in the world by far. The deep sampling viral sequences and the demographic information of all participants provided a solid foundation for further molecular network analysis. For countries with limited sources such as China, HIV drug resistance testing is not routine for all newly diagnosed HIV infections and is usually recommended only when antiviral therapy (ART) fails (Aids et al., 2018). From the view of both the medical care of patients and public health, HIV drug resistance testing should be recommended for all newly diagnosed HIV infections prior to initiation of ART. This is because it helps not only to understand the drug resistance among HIV-infected persons to guide the rational use of antiviral drugs, but it is also of great significance for the construction of local HIV molecular networks to understand local HIV transmission dynamics and to guide prevention and interventions of HIV infection.

The optimized pairwise GD threshold for the HIV molecular network is believed to be very important for better defining transmission clusters (National Center for HIV/AIDS, JUNE 2018). It has been reported that the evolutionary rate of HIV-1 differs across subtypes (Patino-Galindo and Gonzalez-Candelas, 2017), as well as in different epidemiological settings of the same genes (Salemi et al., 2001). For example, the evolutionary rate of HIV-1 subtype A was found to be 8.4 times lower in fast spread among IDUs than in slow spread among MSM (Maljkovic Berry et al., 2007). Therefore, a single GD threshold would likely be unfit for diverse HIV-1 subtypes, especially when these networks were used to target prevention efforts. Three main subtypes (CRF01_AE, CRF07_BC, and B, 93.5%) in Shenyang were the focus of this study and the epidemic situation, and their demographic characteristics were quite different (**Table 1**). We evaluated a GD threshold that would give the maximal number of clusters, i.e., highest resolution of the network with the sequences available. We found that the optimal GD threshold for CRF01_AE and CRF07_BC was distinctly different from that of subtype B and was more conservative than that of subtype B. We also found that similar to the conservative GD threshold (0.005 subs/site), the optimal GD threshold (0.007subs/site) in CRF01_AE and CRF07_BC could be used to identify the higher rate of RHI within defined clusters than the liberal GD threshold (0.015 subs/site) (**Table 2**). The reason may be that they spread quickly and have been the top two prevalent subtypes, accounting for >80% of HIV-1 infected MSM in China (Han et al., 2013). The low GD threshold was close to the criteria for the recent and rapid growth of clusters (0.005 subs/site) provided by Oster et al. (Oster et al., 2018). It was not difficult to understand that the optimal GD threshold for the main subtypes in this study were similar with our previous study with the data collected before 2016 because of the similar HIV epidemics (Liu et al., 2020). Above all, a sensitivity analysis across plausible threshold ranges should be recommended for non-B subtypes before inferring an HIV molecular network. As an alternative strategy, a strict GD threshold (0.005 subs/site) could also be used for inferring HIV molecular network of CRF01_AE and CRF07_BC directly in Shenyang.

Once inferred, molecular networks can identify characteristics of fast-spreading HIV epidemics (Aldous et al., 2012), which can be used to efficiently target prevention efforts. To make the targeted intervention targets more precise, we defined the priority intervention clusters in combination with some risk factors related to active transmission clusters and poor outcomes clusters (National Center for HIV/AIDS, JUNE 2018; Prevention and center, 2019), instead of roughly comparing the demographic information of individuals in and out of clusters. We also utilized transmission rates to estimate the large clusters (size ≥10) to provide targets for intervention which has been previously used to study HIV phylodynamics (Holtgrave et al., 2012). This could be an effective way to prevent new infections by providing more effective interventions in various ways in addition to ART (such as enhanced HIV testing or pre-exposure prophylaxis within identified risk populations or rapid ART or enhanced ART adherence to HIV infections identified within priority clusters (Wang et al., 2015)) to the individuals in the priority intervention clusters. In this study, all eight large clusters were included in the priority clusters because of multiple risk factors. For example, clusters 01AE1, 01AE2, 07BC18, and 07BC20 had both more newly diagnosed HIV-1 infected cases in 2018 (>5 cases) and high TR. Moreover, clusters 01AE2 and 01AE6 contained 80% and 66.7% of IDUs, respectively. Cluster 07BC19 was a TDR cluster with Q58E/V179D. Q58E could confer to low-level resistance to TPV (tipranavir) which is not recommended in China. V179D is a polymorphic accessory NNRTI-selected mutation and may not affect NNRTI sensitivity alone (Rhee et al., 2003). This cluster was identified as priority cluster because of these drug-resistant mutations and the higher transmission rate. Cluster 07BC20 was a TDR cluster with K103N. The latest study showed NNRTI-associated mutations including K103N/S had high percentages in ART-naïve and ART-treated individuals in China because of the currently available first-line ART regimens containing EFV or NVP (Zuo et al., 2020). Phylogenetic analysis showed that strains with K103N were concentrated in the main lineage of CRF07_BC among MSM circulated in Liaoning (**Figure 2**) (Han et al., 2013), and the median age of all individuals in this cluster was only 25 years (range 22-36); six individuals with K103N were diagnosed at RHI stage (75%, 6/8) and this TDR cluster also had the highest TR. These were favorable factors for HIV transmission. This could be the first report of concentrated transmission of HIV drug-resistant strains in China.

These results suggest that the infections in the priority intervention clusters with multiple risk factors might be the important high-risk individuals for HIV transmission in Shenyang and should deserve more attention. Some medium (5<size<10) or small clusters (size ≤5) might also have the potential for further expansion. Due to the limited number of HIV infections in this cluster in a relatively short time, the evaluation criteria of the priority intervention clusters cannot be reached in a timely fashion. According to the proportion of RHI in the cluster (>50%), we can estimate their potential for further expansion and carry out real-time monitoring in the future.

In this study, some age-related clusters were also discovered. Six clusters (01AE5, 01AE9, 01AE10, 07BC19-21) were found to be ‘young clusters’, the median age of which was less than 30 years (median age: 22-27 years), the individuals of which were all male (100%, 63/63), most unmarried (84.1%, 56/63) and had higher RHI rate (median: 57.1%, range 44.4-71.4%) than the average level of all infections. Some studies have demonstrated that the young MSM were among the most important high-risk populations (Balaji et al., 2013; Qin et al., 2017). Interestingly, our results suggested that there were indeed such clusters (cluster 01AE2, 4, 6, 7, 11, 15-17). Their median age was more than 40 years (median age: 44-57 years), some even more than 50 years old; 83.8% (62/74) of them were male, 68.9% (51/74) of them had marriage history, and a higher RHI rate (median: 58.4%, range 18.2-83.3%). These results suggest that elderly men may remain sexually active. A recent large-scale systematic analysis among MSM in China showed there was the highest HIV prevalence (19.3%) in those aged 50 years (Dong et al., 2019), which supported our finding. Among the identified targeted intervention clusters, two TDR clusters (07BC19 and 07BC20) were ‘young clusters’ (median age: 24/25 years) and two IDU clusters (01AE2 and 01AE6) were ‘old age clusters’ (median age: 46/47 years). Taken together, these analyses revealed priority intervention clusters with distinct characteristics such as TDR clusters, IDU clusters, young and old clusters, etc.

## Limitation

There are still some limitations in this study. First, the delay between HIV diagnosis and sequence acquisition could limit network-based intervention. Second, in this study, only baseline information of all infected individuals was analyzed. Lack of more follow-up information including clinical data and treatment information could lead to missing some ART failure individuals. Third, the inferred transmission network from viral sequences does not definitively determine epidemiologic linkage or imply direct transmission. At last, not all the HIV infected individuals could be found and included in this molecular network analysis, therefore, real-world HIV transmission may be underestimated by our molecular network analysis, including the transmission rate of molecular cluster.

## Conclusion

Overall, we identified priority targets for intervention efforts in Shenyang using a comprehensive analysis combining in-depth sampling HIV-1 molecular networks construction and baseline epidemiological information and provided some useful experience regarding using molecular epidemiological methods for complex local HIV epidemics with multiple circulating HIV-1 subtypes.

## Data Availability Statement

The datasets presented in this study can be found in online repositories. The names of the repository/repositories and accession number(s) can be found in the article/**Supplementary Material**.

## Ethics Statement

The studies involving human participants were reviewed and approved by The Institutional Review Board of China Medical University. The patients/participants provided their written informed consent to participate in this study.

## Author Contributions 

HS and XH conceived the study. WS, XD, XL, LW, JL, and HD collected samples and epidemiology data. BZ, WT, and ZW conducted the experiments and collected the data. BZ and MA analyzed the results. BZ drafted the manuscript and XH reviewed and edited the manuscript. All authors contributed to the article and approved the submitted version.

## Funding

This work was supported by Mega-Projects of National Science Research for the 13th Five-Year Plan (2018ZX10721102); The National Natural Science Foundation (81871637); CAMS Innovation Fund for Medical Sciences (2019-I2M-5-027), Scientific Research Funding Project of Liaoning Province Education Department (QN2019005).

## Conflict of Interest

The authors declare that the research was conducted in the absence of any commercial or financial relationships that could be construed as a potential conflict of interest.

## Supplementary Material

Supplementary Figure 1 The molecular network of three main subtypes. a. the molecular network of CRF01_AE. b. the molecular network of CRF07_BC. c. the molecular network of B subtype. Red dots denote the individuals in priority clusters, blue dots denote the individuals in non-priority clusters, the triangle denote injection drug users, and the square denote the individuals with transmitted drug resistance.
