# Supplementary figures and images for: Priority Intervention Targets Identified Using an In-Depth Sampling HIV Molecular Network in a Non-Subtype B Epidemics Area
Source: Front Cell Infect Microbiol. 2021 Mar 29;11:642903. doi: 10.3389/fcimb.2021.642903 (PMC8039375; doi:10.3389/fcimb.2021.642903)

**a**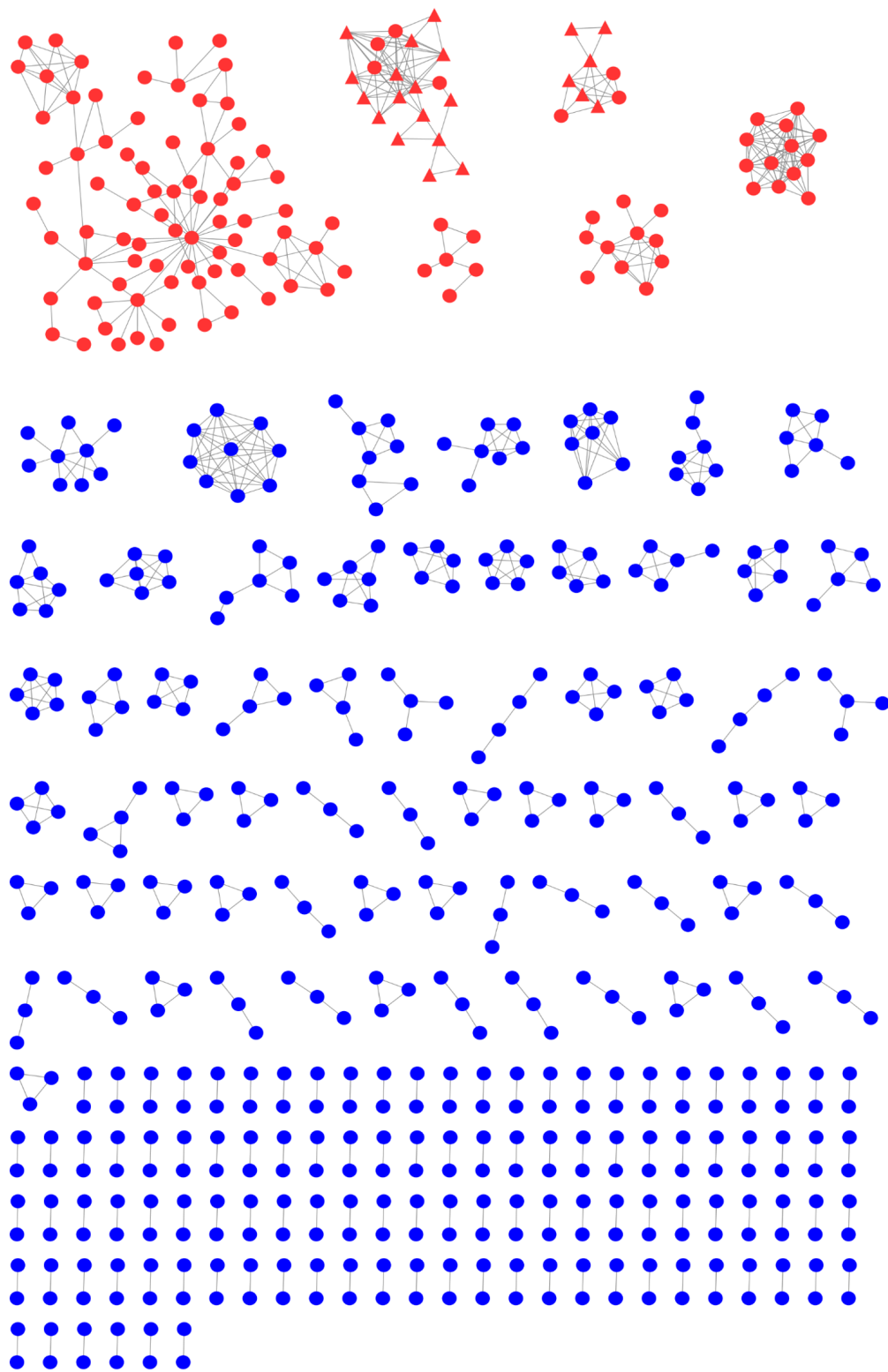**b**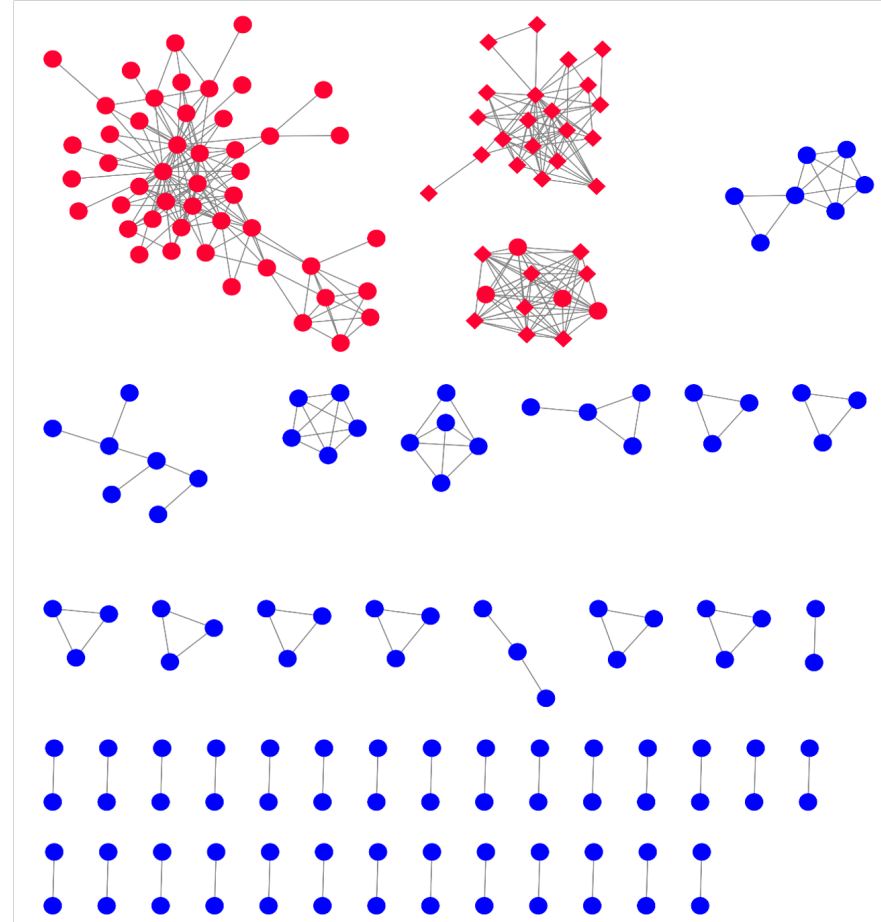**c**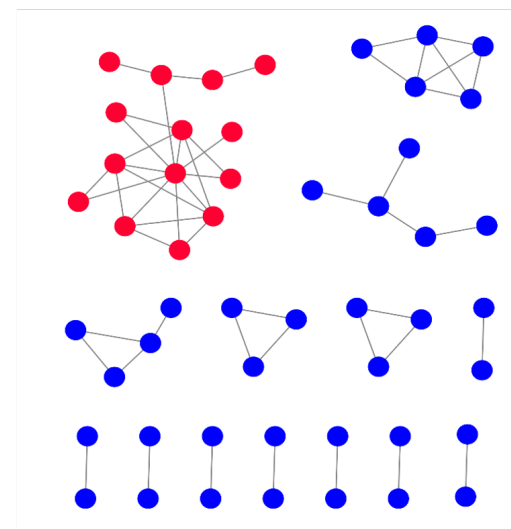

Supplement: Supplementary Figure 1 — The molecular network of three main subtypes. a. the molecular network of CRF01_AE. b. the molecular network of CRF07_BC. c. the molecular network of B subtype. Red dots denote the individuals in priority clusters, blue dots denote the individuals in non-priority clusters, the triangle denote injection drug users, and the square denote the individuals with transmitted drug resistance. [file Image_1.pdf]
